# Intersection between individual, household, environmental and system level factors in defining risk and resilience for children in Kenya’s ASAL: A qualitative study

**DOI:** 10.1371/journal.pone.0316679

**Published:** 2025-01-17

**Authors:** Esther Jebor Chongwo, Barack Aoko, Martha Kaniala, Moses Esala, Phillis Magoma, Eunice Njoroge, Susan Nyamanya, Joyce Marangu, Anil Khamis, John Ng’asike, Anja C. Huizink, Amina Abubakar

**Affiliations:** 1 Institute for Human Development, Aga Khan University, Nairobi, Kenya; 2 Faculty of Behavioural and Movement Sciences, Department of Clinical, Neuro- and Developmental Psychology, Vrije Universiteit Amsterdam, Amsterdam, The Netherlands; 3 University College London-Institute of Education, London, United Kingdom; 4 Turkana University College, Lodwar, Kenya; 5 Kenya Medical Research Institute/Wellcome Trust Research Programme, Centre for Geographic Medicine Research (Coast), Kilifi, Kenya; 6 Department of Psychiatry, University of Oxford, London, United Kingdom; University of Greenwich, UNITED KINGDOM OF GREAT BRITAIN AND NORTHERN IRELAND

## Abstract

**Introduction:**

Children growing up in arid and semi-arid regions of Sub-Saharan Africa (SSA) face heightened risks, often resulting in poor developmental outcomes. In Kenya, the arid and semi-arid lands (ASAL) exhibit the lowest health and developmental indicators among children. Despite these risks, some children grow up successfully and overcome the challenges. However, there is limited comprehensive data on sources of risks and resilience in these children, particularly research that incorporates community perspectives and indigenous knowledge. Systematic documentation of factors influencing child outcomes is crucial for understanding the overall burden, informing policy and implementing targeted interventions. This study aimed at bridging this gap.

**Methods:**

The study was conducted in 10 ASAL counties in Kenya. Purposive and snowballing techniques were used to recruit 11 key informants per site with varied levels of involvement in early childhood development and primary caregivers. Using a semi-structured interview guide, 103 telephonic interviews were conducted between June and August 2022, involving 68 key informants and 35 caregivers. Thematic approach was used to analyze the data, using NVIVO software.

**Results:**

The mean age of the participants was 44years (SD = 11 years). The findings, viewed through the lens of Bronfenbrenner’s ecological systems theory, reveal a complex interplay of contextual factors impacting children’s development. These factors range from individual and biological aspects to family, community, systemic, and environmental level, including climatic challenges. Identified risk factors encompassed issues such as young caregiver’s age, low literacy, mental health issues, drug abuse, domestic violence, malnutrition, poverty, lack of paternal involvement, early marriages, female genital mutilation, drought, heat stress, and insecurity. Nonetheless, sources of resilience include breastfeeding, immunization, responsive caregiving, family and community support, higher socio-economic status (SES), cultural practices, self-motivation, hard work, community role models, religious activities and quality service provision.

**Conclusion:**

Urgent attention is needed to address the multifaceted challenges faced by children in ASAL regions. The study underscores the importance of addressing the root causes of risks while harnessing community strengths and resources to safeguard and promote the holistic development of these children.

## Background

The importance of optimal early childhood development is widely documented. Children attain optimal developmental potential by acquiring essential key skills across physical, cognitive, social-emotional, and language domains, which lay the foundation for lifelong learning, health, and well-being [1]. However, evidence indicates that more than 250 million children, particularly in low- and middle-income countries (LMICs), are at risk of failing to attain their full developmental potential [2]. Children from sub-Saharan Africa (SSA) are vulnerable to developmental delays and disabilities due to a constellation of risk factors, such as poverty and malnutrition, infections, and lack of stimulating home environments [1,2]. Children living in marginalized regions such as the Arid and Semi-Arid Lands (ASALs) face additional vulnerabilities including extreme poverty, chronic insecurity due to conflicts, inadequate access to food, clean water and other basic resources and amenities [3]. However, there is insufficient empirical evidence on the factors that influence child development in this context and especially qualitative work is lacking that takes into consideration perceptions and voices of community members.

ASALs cover roughly 88% of Kenya’s landmass [4]. Although there has been marked improvements in child developmental indicators over the years in Kenya, glaring disparities still exist with children living in the ASAL regions facing a disproportionately higher risk than those from most regions within the country [5]. These regions represent the country’s poorest [5] and contend various challenges such as food insecurity, undernutrition, and climate change effects [6]. Climate change is recognized as a major global health threat, with growing evidence, indicating its adverse effects on young children [6]. For instance, extreme heat and drought because of climate change have been reported to be linked with malnutrition in children due to the associated food insecurity [3,4,7–9]. Other effects reported include reduced breastfeeding, neonatal mortality, still birth, spread of infectious diseases and negative impact on cognitive ability [9].

Children in ASALs often face challenges such as limited access to healthcare services, including vaccinations and treatment for common illnesses, leaving children vulnerable to preventable health issues that can hinder their growth and development [5]. National statistics show that mortality rates for children under-five years mortality rates in Kenya’s ASAL counties can be double the national average [10]. Inadequate sanitation facilities and limited access to clean water are prevalent in ASALs, increasing the risk of diarrhoeal diseases and other infections [11,12]. Socio-economic challenges, including poverty and limited access to resources, create a disadvantaged environment. Furthermore, children in ASAL face limited access to quality education [13], a crucial factor in cognitive and social development [14] which further exacerbates the situation. These and other combined factors create a complex web of challenges that can have a lasting negative impact on child development in ASAL regions.

Despite the various risks that children face there are children who grow up and overcome the challenges. Resilience has broadly been defined as the ability to successfully adapt to adversity and demonstrate positive developmental outcomes despite risks [15,16]. Several protective factors have been documented, ranging from individual factors to family and community factors [17]. Resilient individuals have been found to exhibit positive outcomes in various aspects such as mental health and academic achievement [18]. However, there is limited data available on sources of resilience in children, especially those from ASAL regions in Kenya. Understanding the factors that enhance resilience in children from these communities will be crucial in implementing targeted interventions that can help promote resilience and mitigate the negative impact of the risk factors experienced.

To gain a deeper understanding of the factors, both risk and resilience, that impact child development in Kenya, we utilized the Bronfenbrenner’s ecological systems theory as an interpretative lens [19], as it provides a comprehensive framework for understanding human development. This theory acknowledges the intricate layers of influence that shape a child’s growth and development, emphasizing the complex and dynamic interplay between children and their environment. It advocates for a comprehensive and holistic approach to understanding child development by considering these multiple levels of influence [20]. Additionally, there has been a growing concern to integrate theories, particularly from social sciences, into global public health [21]. Therefore, to fully understand child development, we need to look at how children and various aspects of their environment interact.

The Bronfenbrenner’s ecological systems theory model postulates five interconnected environmental systems: the microsystem, mesosystem, ecosystem, macro system and chronosystem. All these systems collectively exert direct or indirect impact on child development, which explains the various forms of risk and protection. Moreso, there is a continuous interplay of factors across these domains explaining the complexity of factors impacting child development. This theory has been effectively used to study child development in Sub-Saharan Africa, particularly in understanding how various factors across multiple systems influence outcomes. For example, Edburg et al applied the theory to investigate factors contributing to violence against children in South Africa [22]. Similarly, other studies conducted limited resource-settings have used this approach to understand factors affecting child development [23,24] as well as adolescent health and well-being [25–27]. These studies demonstrate the relevance of the framework in examining how interactions across systems shape child well-being in resource-constrained contexts. It underscores the critical importance of fostering nurturing care environments and adopting a multi-sectoral approach to understand the diverse factors influencing child development and to effectively responding to children’s evolving needs.

Building on this model and given the limited research available on the context-specific factors (both risks and resilience) influencing child development in ASAL regions in Kenya, this study aims to qualitatively document the community perspectives on sources of risk and resilience for children residing in these areas. This forms a vital step towards evidence generation and designing contextual strategies and interventions aimed at ensuring that children in these communities not only survive but also thrive, ultimately reaching their full developmental potential.

## Methods

### Study setting

The study was conducted in 10 ASAL counties which are majorly marginalized communities in Kenya. These include Turkana, Isiolo, Mandera, West Pokot, Garissa, Samburu, Lamu, Tana River, Marsabit, and Wajir Counties. These counties are part of the Frontier Counties Development Council (FCDC). These counties have the highest levels of poverty in the country and the lowest development indicators [5].

### Study design and conceptual framework

**Fig 1:**
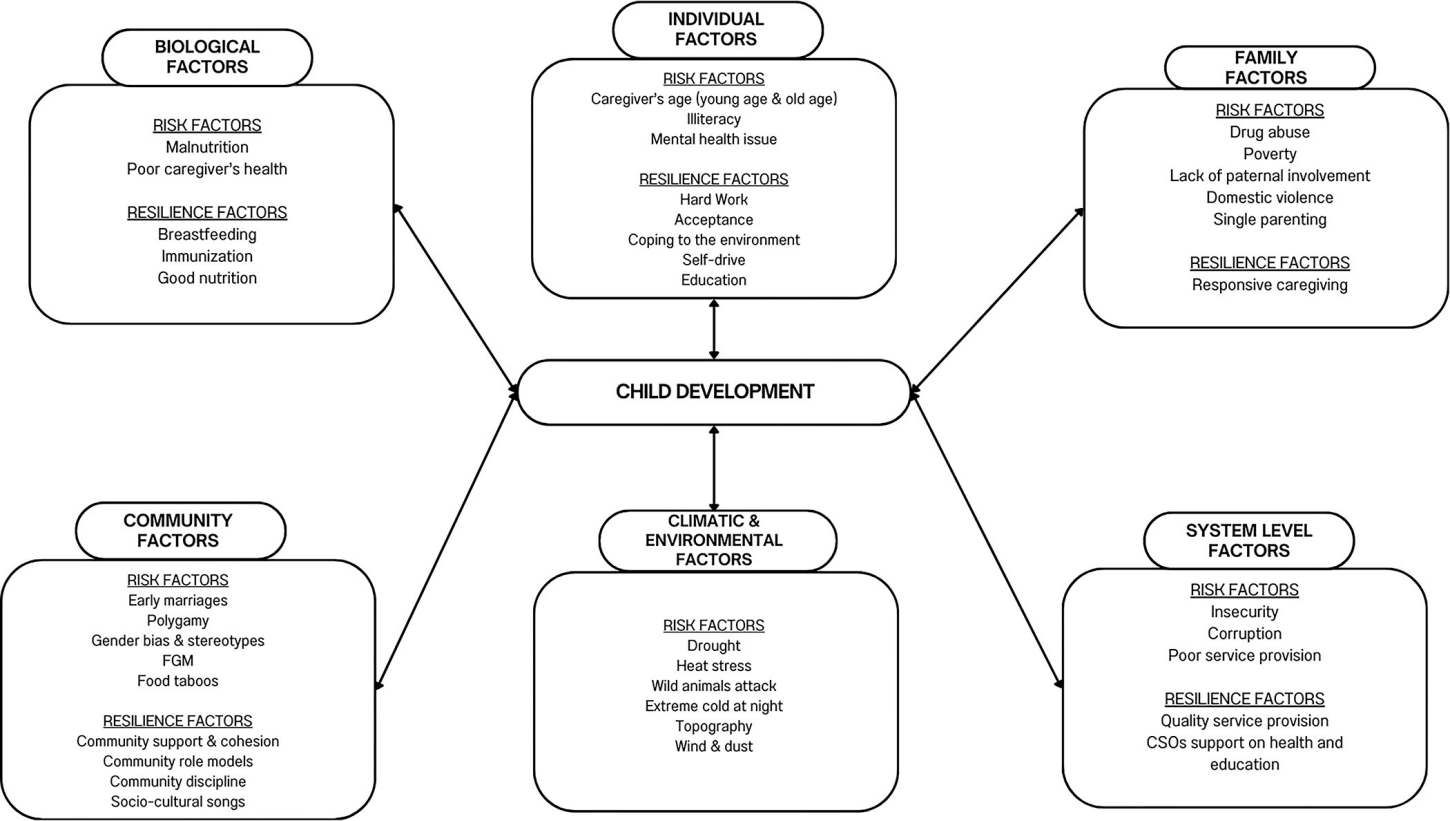
Perceived risks and resilience factors for child development in Kenya’s ASAL.

The study employed a qualitative approach that involved in-depth interviews with key informants and caregivers. A semi-structured interview guide (see S1 File) developed by the study team was administered by well-trained research assistants (BA and MK). We applied the Bronfenbrenner’s ecological systems theory [19] as an interpretive lens to organize and present themes on the factors influencing child development in Kenya’s ASALs. This theory acknowledges the multifaceted layers that impact development. This framework, which emphasizes the layered, interconnected systems shaping human development, guided our analysis across five the key domains: the microsystem, mesosystem, ecosystem, macro system, and chronosystem. Building on this model, we developed a conceptual framework (Fig 1) to guide data collection, thematic mapping and organization of emerging themes during analysis.

### Sampling, recruitment, data collection procedures and management

In total, we conducted 103 in-depth interviews with key informants and caregivers. Purposive and snowballing techniques [28] were used to recruit 11 key informants per site. The selection aimed to include a diverse sample of key stakeholders with varied levels of involvement with the ECD sector. In each site, we recruited 11 key informants, including representatives from the Ministry of Health (n = 1), early childhood education (n = 2)), parents or caregivers (n = 2), social service and gender officials (n = 1), security and local administrative representatives (n = 1), a civil society organizations representative (n = 1), a community focal person (n = 1), disaster management (n = 1), and a religious leader (n = 1). This approach was designed to capture a comprehensive participants perspective across sites.

Following approval from the county, the county directors facilitated the identification of a facilitator who assisted in mobilizing the study participants. The facilitators provided the contact details of selected participants to the research assistants, who then reached out to them in advance to schedule the telephonic interviews at their convenience. Despite having received contact information for 110 participants, seven of them could not be reached by phone and, therefore, could not be included in the study.

As part of the conceptualization work, we reviewed relevant literature to guide the development of the interview guide. The socio-ecological theory guided the development of the interview guide by framing child development within a broader context of interconnected systems. The interview guide was designed to explore factors at multiple levels, including individual, family, community, and environmental influences on child development. By incorporating questions related to these different levels, we aimed to capture the complex interplay of factors that shape child outcomes in the ASAL context.

Data collection was conducted from June 15, 2022, to August 29, 2022. The data were collected by two research assistants (BA and MK) who were well-trained in qualitative interviewing techniques and notetaking. Prior to the commencement of the data collection, we piloted the interview guides with 4 participants (2 key informants and 2 caregivers) to ensure clarity of the questions. Based on the positive feedback received, there were no modifications made to the guide. The data collection was done telephonically and at convenient times to the participants. The interviews lasted between 30–60 minutes. Telephonic interviews were selected because of the significant logistical challenges involved in physical travel to all the 10 vast counties, a decision aimed at optimizing both time and resources. All interviews were conducted either in English or Swahili languages which are the two primary languages used for communication in Kenya. With the participants’ permission, the interviews were audio-recorded, and interview summaries were made after the interviews. Notably, no repeat interviews were conducted. To ensure participant’s privacy and confidentiality the interviews were conducted in private rooms at the workplace and the participants were encouraged to take the call in private and quiet places. Additionally, all the voice files were labelled anonymously using unique participant identifiers. Participants socio-demographic data were also collected including age, gender, religion, residence, occupation, ethnicity, number of children, and the level of education. This information is summarized in Table 1.

**Table 1:** Participants description.

Characteristics	Total (103 participants)
Gender	Males: 58 (56.3%) Females: 45 (43.7%)
Mean age (range) years	43.89 (29–71)
Highest level of education*	Primary and below: 14 (13.6%) Secondary level: 11 (10.7%) Tertiary level: 75 (72.8%) Madrassa: 3 (2.9%)
Religion	Christians: 41 (39.8%) Muslims: 62 (60.2%)
Children	Participants with children: 64 (62.1%) Participants without children: 39 (37.9%)

*Primary and below: Include those who have not gone to any formal school and those with complete or incomplete primary school (from grade 1 to grade 8).

**Secondary Level**: In Kenya, usually 4 years in Kenya. Also called Highschool. Includes those with complete or incomplete.

**Tertiary level:** Tertiary level indicates individuals who have pursued higher education beyond secondary school, including post-secondary vocational training, college, and university education.

Madrassa: Those who had only attended traditionally taught religious studies, such as the Quran, Islamic law and theology.

### Data analysis

All voice files from the interviews were transcribed verbatim in the original languages to ensure accuracy and retention of the original meaning. Subsequently, all the transcripts were reviewed by an independent person, who had not been involved in the transcription (BA, MK, and ME). EJ, BA, MK, ME, PM, EN, and SN collaboratively developed the initial coding framework based on emerging themes, while AA reviewed and approved it. The framework was refined through an inductive approach, informed by initial codes and the original topic guides, allowing flexibility in interpreting participants’ perceptions. The transcriptions were coded in the original language by BA, MK, and ME using a thematic analytic approach [28] in NVIVO QSR software Version 12. Translation to English was performed only for the selected quotes used in the final report. The themes were categorized into (i) sources of risks and (ii) resilience factors, organized based on the bioecological framework. For example, the microsystem includes individual factors such as age, literacy levels, and health status. The mesosystem encompasses family-level factors, including poverty, domestic violence, substance abuse, and parenting practice. The exosystem captures the wider community-level factors such as neighborhood security, political factors like corruption, the role of government, and CSO service provision. The macrosystem involves sociocultural factors such as female genital mutilation, nomadism, polygamy, community cohesion, and cultural support systems. The chronosystem reflects observed changes over time such as climate change effects like drought and overtime decline in some cultural practices. Relevant quotes for each theme are presented in the results section.

#### Validity of results

Several steps were undertaken to ensure the reliability and validity of our research. The study team collaboratively designed the interview guide, piloted it to ensure clarity and effectiveness in eliciting rich data. Experienced RAs conducted the interviews and regular team meetings were held to discuss data collection, emerging themes, and potential biases. Data quality was maintained through team review of the coding framework, interview transcripts and rigorous coding and analysis. Post-data collection and as part of research validation, dissemination meetings were organized, and preliminary results were shared with local stakeholders for further verification of our interpretations and validation, strengthening the research’s credibility. Further, to enhance triangulation, the sample included a diverse group of stakeholders and caregivers from multiple sites, ensuring a broad range of perspectives [29]. Throughout the process, the team paid special attention to their positionality, reflexivity, and subjectivity [30]. By employing these rigorous methodological approaches, the study aimed to ensure credibility and trustworthiness of the findings, aligning with established qualitative research principles [31]. The Consolidated Criteria for Reporting Qualitative Research (COREQ) checklist guided this study’s reporting (see S2 File).

### Ethical considerations

Scientific and ethical approval was obtained from the Aga Khan University’s Institutional Ethics and Review Committee (ISERC) [2022/ISERC_04(v3)] and the National Commission for Science Technology and Innovation (NACOSTI/P/23/17335). Additional approvals were obtained from each of the counties involved. All the study participants provided verbal consent to be interviewed, and audio recorded as the data collection was conducted via telephonic interviews. Following the verbal consent, the interviewer signed a copy of the consent form as evidence and acknowledgement that they adhered to the standard protocols for obtaining informed consent. This process was witnessed by the other research assistant (either BA or ME) to ensure compliance with standard procedures.

## Results

### Participants’ characteristics

In-depth telephonic interviews were conducted among 68 key informants and 35 caregivers aged between 29–71 years (Mean 43.89 (SD) = 10.89 years). There were 60.2% Muslims, and the rest were Christians. Gender-wise, 56.3% were males, while 43.7% were females. Notably, most of the participants had children. See Table 1 below.

### Risk and resilience factors for child development

#### 1. Individual-level factors (micro-level)

*Risk factors*. **Age of the caregiver:** Young maternal age was reported as a significant risk factor for child development. Narratives from participants indicated that young mothers (those under 20 years) face challenges including inadequate resources, limited childcare knowledge, and elevated stress levels, all of which hinder their ability to provide responsive and nurturing care. This caregiving gap often leads to reliance on grandparents for caregiving support, who may also face constraints such as financial hardship or physical limitations, further compromising the quality of care available to children. Early marriages emerged as a central factor perpetuating this cycle, driven by socio-economic pressures and cultural practices that thrust young girls into early marriages and caregiving roles for which they are unprepared. As a result, young mothers are deprived of opportunities for education and skill development, reinforcing cycles of poverty and compounding systemic inequalities that adversely affect child development outcomes. Applying Bronfenbrenner’s ecological systems theory, the challenges associated with young maternal age are not only confined to the individual level (microsystem) but reflects the interconnected influence of other factors such as poverty and socio-cultural norms within the socio-ecological system. A participant described the severity of the situation:

*“Not so much in urban [early marriages], but in rural areas. I will also try to share with you some of the photos I took last week; it’s very challenging. Children having children. We called for a meeting for women and small girls came with children also. So, children are being married as early as 12 years. From 12 years going upwards, they are somebody’s wife. So, it’s a very scary situation.”* ***(Male government official, 43 years)***

**Low literacy:** Low literacy among caregivers emerged as a significant barrier to child development in marginalized ASAl regions. A participant cited, “*I said that there are issues of high illiteracy levels among the major population of Marsabit county* ***(Male government official*, *38 years)*.** Caregivers with low literacy struggle to engage in activities that support their children’s cognitive and socio-emotional development, such as school enrollment, engagement in educational learning activities such as homework and monitoring the child’s progress. They also face challenges in providing responsive care and making informed decisions on healthcare and nutrition decisions and immunizations which affect the child’s overall wellbeing. Literacy levels were thought to be shaped by systemic challenges within the ecological system, such as limited access to educational services. The nomadic lifestyles common in ASALs further exacerbate these challenges, with inadequate mobile learning centers leaving individuals with limited opportunities to enhance their literacy, perpetuating a cycle of inequalities.

*“So, if the mother cannot read, obviously that child is affected in one way or another. In terms of even food preparation, in terms of even understanding the child’s need, in terms of monitoring education at ECD level. The mother has gap in all these areas”.* ***(Male government official, 40 years)***

**Mental health:** Mental health challenges, especially stress, were frequently highlighted as a barrier to effective caregiving. Participants identified various contributing factors including drug abuse, domestic violence, poverty, teenage pregnancies, insecurity, and drought-related effects such as hunger and loss of livelihoods. These stressors collectively undermine a mother’s capacity to provide consistent and nurturing care, which is vital for a child’s healthy development. For instance, stress can lead to increased irritability, impatience, and neglect, impacting the quality of caregiving interactions and perpetuating cycles of neglect and poor developmental outcomes.

*“You know, when a mother is stressed, she will not be concentrating on her child’s affairs, what her child is eating, what she is wearing, whether at this time she is feeling well or unwell because her mental state is not okay. So, stress contributes to the mother not being able to take care of the child”. (* ***Female caregiver, 38 years*** *)*

*Resilience factors*. Individual-level resilience emerged as a key factor in mitigating the impact of adversity on children from Kenya’s ASAl. Key personal traits such as self-motivation, being self-driven, acceptance and coping stood out as key factors contributing to their ability to navigate challenging circumstances. Despite confronting numerous challenges in the ASAL region, these children demonstrate an intrinsic motivation to improve their lives, driving them to endure hardships. Participants emphasized that many children from ASAl regions develop resilience early in life, characterized by their ability to cope, psychological adaptability, and a strong sense of acceptance cultivated through exposure to adversity and harsh living conditions. As one participant noted:

*“Yes, you know despite challenges it’s like this our people have developed resilience. They have that resilience as they are used to this hardship. You know everything from the climate to the geographical setup including insecurity. Everything is a hardship. From an early age, these children are brought up in that way. It is like they have developed a kind of shock absorber. It’s like that resilience in a way. We are used to hardship, so it’s like we have accepted to live with it, so it has become part and parcel of us.”* ***(Male caregiver, 54 years)***

#### 2. Family-level factors (Meso level)

*Risk factors*. **Domestic violence and substance abuse:** Domestic violence and substance abuse were frequently identified as significant risk factors affecting child development. Participants noted that domestic violence profoundly impacts children, causing psychological stress, leading to unresponsive caregiving, particularly from mothers, which further contributes to poor child developmental outcomes. In many cases, domestic violence results in family disintegration which further exposes children to harm. Underlying issues like poverty, drug abuse, and polygamy were identified as contributing factors that exacerbate domestic violence, heightening the risks of neglect and abuse within households.

Similarly, drug and substance abuse were highlighted as rising issues with severe consequences for children. It not only inflicts psychological stress on children but may also normalize such harmful behavior, increasing the likelihood of intergenerational cycles of substance abuse. Participants highlighted the links between drug abuse and domestic violence, with extreme cases resulting in fatalities including child or maternal mortality. Drug abuse further diverts resources from essential needs, leading to malnutrition, impaired growth, and mental health issues, perpetuating cycles of vulnerability and poor outcomes for children.

*“Drug abuse is there. There are fathers who have engaged in drug abuse, and this has made them to completely ignore and neglect their families. There are cases where, because of drug abuse, the father has become hostile in the family they have battered their wives and some of them are even killing their children. On the flip side now, you also find there are a few mothers who engage in drug abuse though they are few and because of that a child will have a problem because this mother will not be able to care for her child as the mother is supposed to care for and love a child. These issues of drugs are becoming a menace now and most of the parents, especially the male parents engage in drugs and consequently making them absentee parents.”* ***(Female caregiver, 54 years)***

**Poverty:** Poverty was consistently reported as a significant risk factor negatively impacting child development at multiple ecological levels. Children from impoverished families are more likely to experience poor health outcomes, including higher rates of illness, malnutrition, and developmental delays, due to limited access to healthcare and educational opportunities. Poverty further exacerbates gender-based violence and contributes to mental health issues, widening social and health disparities across communities. These inequalities further reinforce cycles of poverty, creating significant long-term implications for public health and child development.

*“And then the issue of poverty. When there are no resources at home and the father has gone far away, at times they will go as far as Ethiopia for pasture. So, when women are left alone, without any provision, poverty now takes control and now they have to go round washing people’s clothes to get something to eat, they do petty jobs, and all along I mentioned to you regarding potential loss of dignity. When you go around begging, it now takes away your dignity, because you can be exploited. So, when the woman is in problems it’s like now the child is just restricted…Whenever the mother is in a problem, the child is also in the problem.”* ***(Male government official, 39 years)***

**Single parenting and limited paternal involvement:** Single parenting emerged as a significant challenge, particularly in providing basic needs and ensuring adequate childcare. In most of the ASAL communities, fathers assume the responsibility of tending to the livestock, resulting in prolonged absences from home that can span for years especially during droughts. This absence deprives their children of paternal love, care, affection and emotional support, propagating wellbeing inequalities and disparities in child development. As women are left behind, they face immense challenges in coping with all the responsibilities that fall upon them, often compromising their ability to provide adequate caregiving.

*“You know fathers are busy with the animals. Most of them are away and this negatively affects the children. For example, now for almost three years, we have not received rain. Can you imagine a mother who has a child, and the father has migrated with his animals to other parts of the county like Isiolo or Somali, who is away for eight months from his children and family? So, this kind of drought and these kinds of issues are negatively affecting these children and their mothers.”* ***(Male government official, 40 years)***

*Resilience factors*. At the family level, several factors were reported to contribute to enhancing resilience in children. Responsive caregiving emerged as a central factor, with parents meeting basic needs and offering emotional and practical support to foster stability and growth. High family economic status further enables access to essential resources that enhance positive development. Parental involvement in activities such as discipline, mentorship, and encouragement foster academic success and overall growth. Furthermore, strong intra-family relations create a supportive environment that enhances resilience against diversity. Ensuring supportive and resource-rich family environments can mitigate the negative impacts of broader systemic inequalities and enhance overall outcomes. This aligns with socio-ecological theory, where supportive family environments within the mesosystem influence child outcomes and can mitigate structural inequalities and improve child outcomes.

*“Now about children like for me, as I said if I want my child to be successful. I ensure they go to school; I make sure they have pens and books because that is what will help. For example, if I don’t buy books or pens, I don’t become harsh to the kids and tell them to go to school. I must have my own rules at home, there is no excuse for refusing to go to school. In the evening when they come back home, I check their homework and encourage them to do it; if I fail to follow up on this, children will not succeed.”* ***(Female caregiver, 34 years)***

#### 3. Community and socio-political factors (Exosystem)

*Risk factors*. **Insecurity:** All the ASAL counties reported facing insecurity issues, specifically cattle rustling, banditry and wild animal attacks such as scorpion and snakebites. The sources of conflicts were resource-based, border-based, as well as external attacks from terrorist groups like al-Shabaab. Children were reported to be the most affected, experiencing disruption on learning/education, mental health/psychological stress, displacement and loss of lives. This underscores the exosystem’s influence on child development as outlined in socio-ecological theory, where external environmental factors like insecurity indirectly affect children’s outcomes by disrupting essential services and creating unsafe living conditions.

*“…. You know insecurity brings the issue of displacement, communities will run away from where they live, and you know children are the ones exposed to these things. When they are displaced from the houses or the houses are burnt, first the schools get affected and they’re exposed to so much hazard like for instance wild animals attack because they are outside their houses.”* ***(Male government official, 40 years)***

*“We have had security issues in fact*, *this year alone*, *the last one and half years we have lost over 100 people*. *We have lost over 100 people and the majority of those are school-going children*. *“****(Male government official*, *54 years)***

Interestingly, climate change effects were reported as key drivers of resource-based conflicts particularly due to competition for scarce resources such as water and pasture. These conflicts, often exacerbated by frequent droughts, have profound and far-reaching impacts on children’s lives and well-being.

*“The other hazard we are facing as a country is conflict based, mostly it’s resource-based conflict because of this climate change and this, you know, frequent droughts. Communities are moving from one place to another, and they’re looking for resources, scarce resources in terms of water and pasture. Now, most of these are possible. And so, and the resources they are using are scarce they prone to conflict and they always fight and quarrel over the few resources found around.” (* ***Male government official, 40 years*** *)*

**Political factors:** Political interference and corruption emerged as a significant hinderance to equitable and high-quality in childcare services in Kenya’s ASAL as one responded cited, *“We just have corruption*. *People are there just to make wealth*. *They don’t care about people”* ***(Male government official*, *50 years)*.** Poor governance marked by inadequate planning by both national and county governments exacerbates public health disparities, influencing morbidity, mortality, and long-term child outcomes. Political influence was highlighted to lead to unqualified individuals being appointed to key positions undermining the effectiveness of service delivery.

*“There’s too much interference from politicians who want to allow their people who are not qualified to be given (jobs). Sometimes you cannot even do accountability because they influence the processes and lack of poor planning by the governments or the national government and the county governments to address issues of child development.”* ***(Male government official, 43 years)***

*Resilience factors*. Government initiatives and civil society organisations play pivotal roles in enhancing resilience in ASAL communities through the provision of various support programs and services. These include essential services such as maternal and child health, nutritional support, financing and provision of water and education resources. In the communities, there are civil society organizations complement government efforts by building local capacity and implementing programs such as school feeding schemes, infrastructure development, and emergency response services, particularly during crises like droughts. These programs enhance community resilience and mitigate the adverse effects of systemic inequalities.

*“..There is a resilience program that the county and the partners are doing that is very supportive so with that they can cope up, like when there is no water, the water is just delivered to them, …then integrated outreaches during emergencies. So, during drought we usually improve the access to the health services.”* ***(Female government official, 49 years)***

#### 4. Socio-cultural factors (macro-level factors)

**Female genital mutilation (FGM) and early marriages:** FGM and early marriages, although reduced and illegal, remain prevalent in some ASAL communities posing significant risks to child development. FGM is associated with severe birth complications such as prolonged labor, which can result in developmental disabilities like cerebral palsy due to brain injury. Early marriages, often tied to FGM and dowry practices, force young girls, sometimes as young as 10 years old, into motherhood before they have the physical, emotional, or social maturity to provide adequate childcare. These practices, rooted in the macrosystem and reflect deeply ingrained cultural norms, not only harm maternal and child health but also perpetuate cycles of inequality and limit opportunities for positive developmental outcomes. Below is an account of one of the participants:

*“One of the socio-cultural factors is the FGM (female genital mutilation). FGM is really affecting us directly, especially during delivery. It leads to difficult deliveries and prolonged labor. When the deliveries become difficult it can affect the child leading to cerebral palsy and we term it as a poor score that leads to brain injury. So, you see that outcome is poor.”* ***(Male government official, 39 years)***

**Nomadism:** Nomadic way of life by many residents in ASALs further exacerbates challenges to child’s welfare by disrupting education and access to early learning opportunities. Frequent relocations for livestock care, especially during prolonged droughts, hinder school attendance and enrollment. This lifestyle also disrupts paternal involvement due to extended absences from home placing significant strain on maternal well-being. Participants reported that when women are left behind, they face exploitation and poverty which further affects their children’s welfare.

*“…Most people in Isiolo are herdsmen. They take care of livestock and sometimes you see some students are forced to go and take care of goats and sheep. They become late to start schooling because they are always there with livestock and their parents say education has no value, the livestock will help them. So, there is a problem on that side.”* ***(Male government official, 52 years****)*

**Polygamy:** Polygamy, though culturally accepted in many ASAL communities, some participants cited its potential negative effects. It was thought to contribute to maternal stress, neglect of parental responsibilities, domestic violence, and heightened vulnerability during resource-scarce periods such as drought and famine. The strain of managing multiple households was noted to lead to neglect of children’s needs and higher rates of divorce, impacting children’s emotional and maternal well-being. A male caregiver explained:

*“You find most of the men here, the culture is polygamous, so you find that despite somebody’s financial limitation they end up marrying two to three wives. So, he may not have adequate time or resources to provide for all these children so in the process he can end up ignoring some of the children and some of the wives. And sometimes they may see divorce as solution because they like running away from their responsibilities, so unstable marriages are one of the issues that can actually lead to child not having the needs.”* ***(Male caregiver, 54 years)***

*Resilience factors*. **Socio-cultural norms**: Interview accounts indicated that socio-cultural norms, like the practice of "traditional maternity leave," emerged as key resilience factor supporting maternal and child well-being. It facilitates ample recovery time for mothers post-delivery, allowing them to adequately focus on their recovery and care for their babies. During this critical moment, comprehensive care is extended by community members, with a focus on nutrition and the overall well-being of the mother and child. Additionally, communal cultural activities such as songs and dances further not only strengthen social bonds but also promote talent development, contributing to children’s socio-emotional development.

*“We have actually what we call maternity leave for about 40 days after this lady gives birth so that she can have milk, and she can start expressing. So, we have a lot of social responsibility within us, and we are very particular about our children.”* ***(Male government official, 67 years)***

**Community support and cohesiveness:** The strong sense of shared responsibility and mutual support within these communities was noted to create a protective environment for children. As reported, resource-sharing and collective care practices, common in ASAL communities, contribute to children’s safety and well-being, mitigating the effects of socio-economic disparities. This sense of collective responsibility is exemplified in the following quote:

*“You know the community supports one another. There is community support… If you see some children playing in a dangerous place or fighting, the community helps. If you have bought a book and your kid has graduated from that grade, you help your neighbor, without even selling it to him. If a household is food insecure and they have not eaten, the neighbor or the relative who has something will also chip in and they will share the little they have.”* ***(Male government official, 43 years)***

**Religious factors**: Religious beliefs and practices play a crucial role in enhancing coping and resilience among children. Faith-based organizations play a critical role in addressing vulnerabilities in the ASAL region through provision of essential support such as education, feeding programs, moral guidance and opportunities for learning. For example, Islamic programs like the Madrassa and Duksi provides children with religious education, instill moral values, offer social support and a sense of discipline, fostering resilience and identity among children.

*“We have the interfaith, faith-based organizations that are sponsoring kids, training good wishes of religion in schools, and building their faith which has molded children into responsible citizens…They are integrating religion or faith into the everyday life of a child, you know, that person become a responsible citizen.”* ***(Male, government official, 38 years)***

#### Climatic and environmental factors (chronosystem)

**Drought:** The impact of drought in the ASAL was perceived to be multifaceted with significant effects on various aspects of child development. Drought was reported to exacerbate malnutrition due to food insecurity arising from the drought-related effects. The scarcity of water during droughts also compromises hygiene and sanitation practices, increasing the risk of waterborne diseases such as cholera. The lack of resources also disrupts children’s education, as families struggle to meet basic needs.

*“You know in our county drought is the major one because with drought we don’t have good enough water we don’t have good hygiene we don’t have food production. It causes almost everything.”* ***(Male caregiver, 63 years)***

*“Like now when we talk of drought when the livelihood is affected the animals are dead which the family depends on then that already can affect the food security of a household*, *and it can bring marasmus or kwashiorkor or any other malnutrition-related diseases*.*”* ***(Male government official*, *53 years)***

***Heat stress*:** Heat stress was identified as one of the major effects of climate change with numerous effects. It was reported to cause dehydration, skin diseases, blood pressure fluctuations, and fatigue, as well as miscarriage and challenges in breastfeeding for pregnant and nursing mothers. The strain from heat also affects productivity and learning and concentration of young children, especially those learning under trees and poor structures. Pregnant mothers face further difficulties, including discomfort and sleep disturbances, which can negatively affect their health and the development of their unborn children. Other participants expressed that extreme heat could affect a child’s brain development and exacerbate disease-sensitive diseases.

*“In a number of ways, sometimes heat rash. You can see children, although I am not an expert in health, but you can see sometimes children having rashes, you can see children being dehydrated. And so, they need water frequently. You can even see their own parents losing that breast milk to feed the kids due to high heat. Then you know kids will be susceptible to diseases and then in this high temperature, we have high cases of malaria due to mosquitoes and stuff like that. So, heat affects children in a number of ways.”* ***(Male government official, 38 years)***

**Floods:** Although floods were mentioned to be infrequent, their occurrence brought about substantial disruptions on children’s health and education. Participants noted that floods cause displacement, increase risk of waterborne diseases and disrupt children’s education.

*“For the children sometimes all their schools are flooded and then they become IDPs (internally displaced people).”* ***(Female government official, 26 years)***

"*There are others like floods which when they come*, *they come with diseases such as cholera*, *diarrhoea*, *and many others*.*"* ***(Male government official*, *53 years)*.**

### Cross-cutting theme (Biological factors)

**Malnutrition:** Most participants emphasized that malnutrition is a critical issue affecting early childhood development in the ASAL counties. The primary causes of malnutrition identified were multifaceted, including poverty, drought, food insecurity, poor maternal feeding, limited knowledge about proper feeding practices, and cultural food taboos. These factors, shaped by broader socio-economic and environmental inequities, exacerbate disparities in child health and development outcomes. The consequences of malnutrition were reported to be extensive leading to sub-optimal child outcomes including increased susceptibility to diseases, poor physical growth and stunted development. Moreover, malnutrition impairs cognitive development affecting children’s learning abilities, educational progress, and future opportunities which could contribute to cycles of inequality across generations.

*We have a lot of challenges when it comes to food. If you visit Marsabit today, you will find a child who is 7 years old but resembles a 3-year-old child or a 4-year-old child because of (lack of) food.* ***(Male caregiver, 46 Years)***

Notably, breastfeeding supplementation, and immunization stand out as key biological contributors to resilience. Breastfeeding not only provides essential nutrients and antibodies but also fosters a strong maternal bond and provides critical protection against health issues, laying a solid foundation for children’s resilience and overall well-being. The provision of food supplements becomes essential, particularly when maternal nutrition affects milk production. These supplements, enriched with vital vitamins and protective minerals, ensure optimal nourishment, promoting growth and well-being. Similarly, immunizing children below the age of five was noted as a critical public health strategy aimed at enhancing children’s immune systems, providing protection against potential health threats, and reducing mortality risks.

*“Vaccinating children below five years also boosts their immune system. Others are also providing food supplements because their mothers are not producing enough milk because they are not eating well, so they are provided with supplements that have stronger vitamins components and other protective minerals. Again, responsive care of the mother to the little one and exclusive breastfeeding.”* ***(Male government official,* 43 years)**

*Summary of the perceived sources of risks and resilience for child development in ASALs*. **S1 and S2 Tables** provide a detailed summary of the perceived sources of risks and resilience. The study participants revealed a range of risk and resilience factors affecting child development. Drawing from Bronfenbrenner’s ecological systems theory, these factors manifest at various levels, notably the individual, family, community, environmental, biological domains, and system level. These factors seem to be interconnected across various levels. For example, malnutrition, a biological risk, was attributed to a complex interplay of factors including environmental and climatic changes, poverty, lack of knowledge and food taboos among others. Notably, sources of risks and resilience for child development were evident across all the levels which point to the need to enhance the protective factors and reduce the risks. **Fig 1** below summarizes this information.

## Discussion

This study aimed to examine the risks and resilience factors influencing child development in Kenya’s arid and semi-arid regions using a qualitative study design. The data revealed a complex interplay of factors that may impact children’s well-being ranging from biological, social, environmental, climatic, and psychological factors, highlighting disparities in health and well-being across ASAL regions. The bioecological framework highlights that child development is shaped by daily experiences through ongoing reciprocal interactions between the individual and their environment [32]. The interplay of these factors underscores the interconnectedness between ecological systems and the multi-dimensional nature of child development. These findings underscore the urgent need for targeted multi-level interventions that consider the dynamic interplay between these factors across different levels to address the challenges faced by these vulnerable populations.

Malnutrition was identified as a critical issue affecting early childhood development in the ASAL regions. There is consistent evidence that malnutrition continues to be a major public health problem throughout the developing world, particularly in developing countries such as those in sub-Saharan Africa [33]. The consequences of malnutrition, as reported by our participants, are consistent with findings from the literature. Participants reported that malnourished children are more susceptible to infections and illnesses due to a weakened immune system which can further impede their overall development and well-being [34,35]. Poor physical growth and stunted development were also common outcomes of malnutrition which have been previously documented to be linked to adverse outcomes, such as poor developmental and clinical outcomes [36,37]. Moreover, malnutrition was reported to be associated with cognitive impairment [38,39], affecting children’s learning abilities, hindering their educational progress and future opportunities.

Malnutrition in ASALs was identified as a complex issue with multifaceted causes across various ecological levels (individual, family, community, systemic and climate levels). Poverty, a key family-level factor, that limits access to food, was reported as one of the facilitators for poor outcomes which has been documented in previous studies [40]. Climate-related factors such as drought worsen existing poverty and contribute to food insecurity which further exacerbates malnutrition [3,4,7–9]. Cultural practices including food taboos further restrict dietary diversity and affect nutritional outcomes. The interconnectedness of these factors reveals the deep-seated, multi-dimensional nature of malnutrition in ASAL counties, underscoring the structural determinants of health and the systemic inequities that perpetuate disparities within the country. For instance, data from the KDHS highlight significantly poorer nutritional outcomes in ASAL regions compared to the national average [5]. Addressing malnutrition in these regions would require a holistic approach that encompasses poverty alleviation, education, healthcare, and cultural sensitivities.

In this study, maternal age was reported to influence child outcomes in various forms, aligning with previous research findings [41]. Participants highlighted the perception that majorly young maternal age could potentially pose risks to child development. Young mothers were believed to face a higher likelihood of pregnancy or birth complications and may also lack the capacity to adequately care for their children, with potential negative effects on maternal mental health [42]. A few participants suggested that advanced maternal age could also have negative effects on the children they may have reduced the capacity to provide adequate childcare, often due to health-related concerns, such as age-related illnesses. Comparable studies conducted elsewhere have reported similar findings [43]. Within the ASALs, poverty and socio-cultural factors such as early marriages, are some of the key drivers for teenage pregnancies. Therefore, there is a need to reinforce measures aimed at preventing early marriages through awareness campaigns, community outreach, and legislative reforms to improve overall outcomes. Additionally, comprehensive programs empowering young women with education, economic opportunities, and reproductive health knowledge can potentially address these challenges and contribute to overall well-being.

Furthermore, maternal mental health was reported to play a role in child outcomes. Stressed caregivers were reported to be unresponsive to their children’s needs. Caregivers have been reported to perform key tasks which influence a child’s growth and development [44], and when they are not mentally stable, they were reported to neglect child care duties and not adequately stimulate their children. Early interaction between the caregiver and the child is incredibly important in creating a stimulating learning environment [45]. Maternal mental health can affect childbearing behaviours and practices [46]. Caregiver’s depression presents a substantial developmental risk in children as they are dependent on their caregivers for care [42]. Empirical evidence suggests that children of caregivers with depression demonstrate a range of neurocognitive, sociological, emotional, and behavioural issues [47]. Various factors across various levels interact to exacerbate mental health issues.

Domestic violence was reported to be common in ASALS and has been consistently reported to lead to poor outcomes not only for the mothers but also for the children [48]. Some of the drivers of domestic violence such as drug abuse and poverty were also noted as key factors that lead to impaired child development. Illiteracy is also very high in these communities which was also reported as a risk factor as it limits their knowledge capacity to take good care of the child and also health-seeking patterns [49]. According to KHDSS 2022 [5], the ASAL regions in Kenya have lower literacy levels compared to the national average rates which is attributable to various issues including early marriage, poverty, teenage pregnancies and limited access to resources among others. Inadequate paternal involvement also emerged as theme impacting on child development which in these communities is attributable to various factors including nomadism where fathers go for long time in search of the pasture during the droughts. Additionally, single parenthood which was reported to be caused by various factors including insecurity remains a challenge. Data show that paternal involvement leads to higher socio-emotional, behavioural and cognitive development [50–52], hence children lacking it may have poorer outcomes.

Our study provided valuable insights into the multifaceted impact of climate change on child health outcomes in the ASAL counties aligning with existing literature on climate change and child health [53–55]. Key informants in this study emphasized the presence of several climate change-related extreme weather events, notably including droughts, floods, and extreme temperatures, such as heat stress and extreme cold [56,57]. Drought, in particular, emerged as a complex challenge with far-reaching consequences, consistent with studies documenting the adverse effects of drought on child health [57]. It contributes significantly to food insecurity and subsequent malnutrition among children which is common in the ASAL communities, disrupts education due to a lack of essential resources and compromises hygiene (WASH), increasing the risk increasing the vulnerability of children to waterborne diseases [4,55].

Extreme heat (heat stress), another major climate change effect was reported to cause diverse health effects including dehydration, fatigue, headache, skin disorders, malnutrition, blood pressure fluctuations, and complications during pregnancy and breastfeeding [56,58–60]. The association between high temperatures and reduced productivity and increased fatigue affects both children and their caregivers [56,61]. Importantly, heat stress also poses a cognitive challenge, as elevated temperatures can potentially impact a child’s brain development [62]. Beyond these immediate effects, our study also revealed the cascading effects of climate change which extend beyond their immediate health consequences. Mothers, who often in the ASAL are responsible for fetching firewood and water face physical strain and discomfort, especially during pregnancy, due to the necessity of traveling long distances for water and resources. Moreover, the competition for scarce resources, intensified by climate change-induced resource scarcity, further exacerbated insecurity and instability, significantly impacting children’s well-being. These findings contribute to a deeper understanding on the dynamics of climate change child health in ASAL and underscores the need for addressing these multifaceted challenges through comprehensive strategies encompassing climate resilience, healthcare provision, education support, and conflict mitigation.

Our study also revealed the socio-cultural factors significantly shaping early childhood development in the ASAL counties encompassing FGM, early marriages, polygamy, food taboos, and nomadism which altogether underscore the complex relationship between cultural practices and child well-being in this region. FGM, despite being illegal and acknowledged for its detrimental effects, persists in certain communities, posing great risks to girls and women [63,64]. The consequences extend to birth complications, endangering the health of both mothers and children. Similarly, early marriages remain a concerning issue, particularly in rural/interior communities, where some children are married off as young as 10 years old. These marriages are often tied to dowry payments and the persistence of FGM, leading to birth complications and compromising child development [48]. Addressing these issues requires a critical public health approach to tackle structural inequities that perpetuate FGM and early marriages, emphasizing education, community empowerment, and enforcement of laws to curb harmful practices.

Despite the myriad challenges that children may encounter, there are those who not only persevere but thrive, demonstrating remarkable resilience [65,66]. One of the central pillars of resilience observed in our study is the significance of responsive parenting and robust family support systems [65,67]. Children thrive when they receive consistent emotional support and have their basic needs met. Parents and caregivers who are actively involved in their children’s lives play a pivotal role in nurturing resilience [68]. Responsive caregiving not only ensures the physical well-being of children but also fosters emotional security, which is essential for coping with adversity [69]. Our findings emphasize the importance of the parent-child relationship in building resilience and underscore the need to empower caregivers to improve developmental outcomes.

At the community level, several factors were highlighted to foster child resilience in ASAL counties including communal support, socio-cultural practices, and shared responsibility. Cultural norms, such as the practice of "traditional maternity leave," ensure that mothers have sufficient time to recover after childbirth and allows them to provide adequate care for their infants and enhance the well-being for both mother and child. The ASAL communities in Kenya have active participation in cultural activities, including songs and dances, fosters a positive environment that encourages talent discovery and development among children. Moreover, the sense of community cohesiveness and mutual support within these regions leads to the practical sharing of resources and interventions to safeguard children from harm [70]. This shared responsibility alleviates various challenges faced by families. Despite the decline in communal discipline, it remains a distinctive feature of community-related factors. It signifies the collective effort to guide and nurture children into responsible citizens, imparting moral teachings and serving as a source of inspiration and motivation. Understanding the role of these factors is essential for designing culturally sensitive interventions that enhance child development.

Religion was also a key theme reported to be a significant source of resilience for children in ASAL areas. Both Islamic and Christian faiths provide moral guidance, educational support, and a sense of purpose, contributing to the overall well-being and resilience of children in these communities [71]. These faith-based organizations, actively engage in relief efforts especially during droughts, build boarding schools tailored for pastoralist communities, and sponsor programs to support these children. In the Islamic culture, the Madrassa and Duksi programs provide children with religious education, impart moral and ethical values, offer social support, and instil a sense of discipline. Early exposure to the programs where children learn to read and write in Arabic and memorize religious texts like the Quran, was seen as beneficial to the children’s cognitive development. However, a challenge was identified that most young children in Muslim communities within ASAL areas attend these Duksi and Madrassa programs until later ages, potentially delaying or missing out on early childhood education. This highlights the need to integrate these programs into the formal education curriculum to ensure a holistic approach to early learning and address existing educational inequalities. Recognizing the positive impact of religious institutions is vital for designing holistic approaches to support children’s development and resilience in these unique regions [72].

Furthermore, our study highlighted the critical role of biological factors in enhancing resilience among children in Kenya’s ASAL regions. Specifically, immunization and nutritional support, breastfeeding, emerge as pivotal contributors to children’s resilience. Immunization programs targeting children under five are essential interventions that fortify their immune systems, providing robust protection against various health threats. This proactive safeguarding of their physical health not only reduces the risk of illnesses but also strengthens their overall capacity to withstand adversity and enhance future outcomes [73]. Furthermore, in those experiencing food insecurity, nutritional support, including the provision of food supplements, proves vital. These supplements, enriched with essential vitamins and protective minerals, ensure optimal nourishment, consequently promoting growth and overall well-being in children. Moreover, breastfeeding was identified as a practice that significantly enhances child outcomes and resilience [74–76]. Beyond its nutritional benefits, breastfeeding fosters a strong maternal-child bond, supports cognitive development, and emotional security, and serves as a protective measure against various health issues, thereby laying a firm foundation for a child’s resilience [74,77].

Drawing on the socio-ecological theory, this study highlights the complex interplay of factors (both risks and resilience) affecting child development, extending beyond individual-level factors to include the wider determinants of health. The multifaceted risk factors and systemic inequities observed in ASAL regions resonate with the broader global health challenges, emphasizing the critical role of structural determinants and the need for interdisciplinary approaches that integrate social science perspectives to achieve equitable public health outcomes [78]. Hence there is need to contextualize the children’s development within their ecological system to understand the structures the barriers and enablers that influence their ability to thrive, underscoring the need to implement health promotion programs targeting the wider determinants of child health and wellbeing [79]. Additionally, there is a need to identify synergies and leverage intermediaries across various levels within the ecological system to facilitate the implementation of health promotion programs.

Building on these insights and drawing from the results of this study, we propose some practical actions to address the challenges facing children in Kenya’s arid and semi-arid regions (ASALs). To improve child developmental outcomes, a comprehensive multi-sectoral approach is needed. Interventions should target health, nutrition, education, families, social protection, responsive caregiving, and climate resilience [80]. We propose programs and interventions targeting the reduction of malnutrition that address the root causes such as poverty alleviation, food taboos, and food insecurity, particularly in the context of climate change [80]. Addressing these challenges requires comprehensive policies that go beyond individual responsibility and tackle structural determinants of poverty, as La Placa et al critiques neo-liberal approaches to parenting [81]. Policies should prioritize reducing material deprivation, promoting equitable access to resources, and fostering supportive community networks to empower caregivers and improve child outcomes in ASAL regions. For example, comprehensive nutritional programs should be developed to address malnutrition, complemented by strengthened healthcare access to provide essential services and support for caregivers. Implementing multi-dimensional poverty alleviation programs such as social safety nets, economic empowerment, diversification of livelihoods, supporting small-scale businesses, and enhancing resource governance will be key to reducing vulnerability and improving outcomes [82,83]. There is evidence that effective investment in early childhood development can help mitigate the negative impacts of poverty, malnutrition, and limited learning opportunities, reducing inequalities and promoting equitable development [84].

Furthermore, there is a need to strengthen community–based primary healthcare to enhance maternal and child health services [85]. These services should integrate mental health support into routine maternal care to improve overall maternal and child health outcomes [86]. The study also highlights the importance of legislative reforms and community-based interventions to prevent early harmful cultural practices such as FGM and early marriages. Additionally, policies should strengthen community resilience by fostering communal support systems and recognizing the positive role of religious institutions and families in promoting child development [87,88]. It is essential to incorporate indigenous knowledge and practices in the co-creation of context-specific interventions. Interdisciplinary engagement and investment in climate resilience measures is essential to mitigate the impacts of drought, floods, and extreme temperatures. This involves promoting sustainable agriculture practices, improving water management, increasing funding on climate and health programs, and building climate-resilient infrastructure [89]. Furthermore, there is need to incorporate indigenous knowledge and practices in the cocreation of contextual interventions. Continued research and monitoring are essential to track progress, identify emerging challenges, and inform policy adjustments. This will ensure that interventions are evidence-based and responsive to the evolving needs of children in ASALs.

## Conclusion

Children residing in ASAL counties confront a complex interplay of factors at different socio-ecological layers that significantly impact their development including, insecurity, socio-cultural factors, malnutrition, domestic violence, drug abuse, mental health issues, poverty, and climate-change-related factors like drought and heat stress. Despite the risks, some resilient children overcome the challenges and grow up successfully. Effectively addressing these challenges, many of which are modifiable, requires targeted and holistic interventions that adopt a comprehensive multi-sectoral approach. This involves leveraging community strengths and resources drawing upon community support, positive socio-cultural practices, and tapping into the positive influence of religious factors to enhance children’s overall development and well-being. To mitigate the impacts of climate change, there is a need for investments in community climate resilience. Adopting a multi-sectoral strategy that integrates health, education, social protection, and climate resilience measures will be key to addressing the complex challenges faced in the ASAL regions, ultimately improving child development outcomes. These efforts align with the global commitments outlined in the Sustainable Development Goals (SDGs). Further research is needed to quantify the impact of these factors, including longitudinal studies to evaluate the effects of heat on maternal and child health.

## Strengths and limitations

The qualitative approach enabled us to collect rich and detailed data on the experiences and perceptions of participants. By conducting interviews with a diverse sample of key stakeholders in the ECD sector across various ASAL regions, we captured a wide range of perspectives and deep insights into the factors affecting child development in ASALs. The systematic data collection and analysis approach further strengthened the study’s, ensuring rigorous and reliable findings. While telephonic interviews provided flexibility and convenience for data collection across various study regions, we acknowledge several limitations. The absence of face-to-face interactions may have resulted in missing non-verbal cues that could have enriched participants’ responses. Additionally, we were unable to reach 7 participants despite repeated call attempts and in some instances faced network challenges that affected the clarity of some audio recordings, potentially affecting data quality. While telephonic interviews offered flexibility, they may have limited our ability to observe non-verbal cues, potentially affecting data richness.

## Supporting information

S1 File Interview guide. (DOC)

S2 File Consolidated criteria for reporting qualitative studies (COREQ): 32-item checklist. (DOCX)

S1 Table Supplementary table 1 on sources of risks. (DOCX)

S2 Table Supplementary table 2 on sources of resilience. (DOCX)
