# Supplementary material for: Intersection between individual, household, environmental and system level factors in defining risk and resilience for children in Kenya’s ASAL: A qualitative study
Source: PLoS One. 2025 Jan 17;20(1):e0316679. doi: 10.1371/journal.pone.0316679 (PMC11741590; doi:10.1371/journal.pone.0316679)
Supplement: S1 File — (DOC) [file pone.0316679.s001.doc]

**S1 File. Interview guide**

**(English and Swahili)**

Consent and practical instructions

| **Consent process**  Greet the participant.  Introduce yourself.  Give background information about the study. Carefully read through the informed consent form and answer any questions. Give information about the interview.  Assure the participants that **they do not have to participate if** they do not want to.  Ask for **approval** to participate in the interview.  Tell the participants that s/he can stop participating in the discussions any time they wish and assure privacy and confidentiality.  Explain to the participants that the interview will be recorded and seek approval. Explain the **purpose of recording**.  Ask if there are any questions, after addressing all questions ask for consent to participate in the interview and consent for recording.  At the end of interview collect the information in the data capture form   - **NOTE: Do NOT** record respondents name or any identifier in the data capture form - NB: Start with climate setting |
| --- |

***AIMs****:*

i) To document the risk factors for child outcomes (maternal factors, paternal factors, socio-cultural factors, climatic and other environmental factors, etc.)

i) To identify the sources of resilience and coping for children and caregivers within these marginalized ASAl communities

1. As we start, I would like to know your general experience in taking care of children? (A question for caregivers)

Tunapoanza ningependa kujua kwa ujumla uzoefu wako katika kutunza Watoto? (Umelea Watoto wangapi)

1. As we start, I would like to learn more about what your work involves in relations to early childhood development. (Manly asked to the government officials)

*Tunapoanza ningependa kujua zaidi kuhusu kazi yako inavyohusiana na malezi ya watoto.* ***(***Katika utafiti wetu ECD inamaanisha kipindi mama anapata mimba hadi wakati mtoto anafikia miaka mitatu)

1. What challenges do you face while raising your children *(probe in terms of access to* health, adequate nutrition, security and safety, opportunity for early learning, and responsive care giving-adequate time to take care of the child, ability to respond to child needs and paternal support.

Ni changamoto zipi unazopitia katika malezi ya watoto ( sisitiza katika masuala ya kupata huduma ya afya, lishe bora , ulinzi na usalama,fursa ya kujifuza mapema na kutoa malezi bora )

3. What are the factors that may contribute to poor childhood outcomes in your community. Probe;

a) Maternal factors

b) Paternal factors

c) Socio-cultural factors (traditional beliefs and practices child rearing)

d) Climatic and other environmental factors

e) What are some of the effects of heat stress on maternal and child health (pregnancy, newborns and young children)

Ni mambo gani ambayo yanaweza kuchangia watoto wasikue vizuri katika jamii /matokeo duni kwa malezi ya watoto katika jamii yako (sisitiza)

1. Changamoto za mama (mafadhaiko/ dhiki, miaka, afya, kiwango cha elimu,unyanyasaji wa nyumbani)
2. Changamoto za baba( kukosa usaidizi,baba wasiokuwa,matumizi ya dawa za kulevya)
3. Mila na desturi za jamii zinazohusiana na malezi ya watoto.
4. Hali ya anga na mambo mengine ya mazingira (kiangazi, mafuriko,joto na mafadhaiko)
5. Nieleze athari za joto jingi kwa afya ya wamama wajawazito na watoto (wale wamezaliwa na watoto wadogo).
6. Although children from the ASAL areas face greater risk/challenges, there are also those who adapt to the situations and grow up successfully. In your opinion, what are some of the things that enable these children have positive outcomes despite the risks i.e to be resilient (probe; existing practices that enhance children’s wellbeing in this community e.g family factors, community factors, individual factors, socio-cultural factors)

Ingawa watoto kutoka maeneo yenye ukame wanakabiliwa na hatari/changamoto nyingi, pia kuna wale ambao hustahimili na kustawi . Kwa maoni yako , ni mambo gani yanayowawezesha watoto hawa kuwa na matokeo mazuri licha ya hatari zinazowakumba kwa mfano kuwa wakakamavu/wastahimilivu (mazoea yaliyopo ambayo yanaboresha ustawi wa watoto katika jamii hii -sisitiza mambo ya kifamilia, mambo ya kibinafsi,mambo ya kijamii na kitamaduni.

1. In your own opinion, what are some of suggestions or recommendations for ensuring children grow well in your community?

Kwa maoni yako, ni mapendekezo yapi yatakayowezesha watoto kupata malezi bora katika jamii yako?

**Conclusion**

We have come to the end of the discussion. Any additional comments or suggestions?

**Mwisho**

Tumefika mwisho wa mazungumzo yetu.je una maoni au mapendekezo yoyote ya kuongezea?

***Thank participant for their time and contribution and end recording***

Shukuru mshiriki kwa muda na mchango wao na kumaliza kurekodi
