# Supplementary material for: Intersection between individual, household, environmental and system level factors in defining risk and resilience for children in Kenya’s ASAL: A qualitative study
Source: PLoS One. 2025 Jan 17;20(1):e0316679. doi: 10.1371/journal.pone.0316679 (PMC11741590; doi:10.1371/journal.pone.0316679)
Supplement: S2 File — (DOCX) [file pone.0316679.s002.docx]

**S2 File: Consolidated criteria for reporting qualitative studies (COREQ): 32-item checklist**

Developed from:

Tong A, Sainsbury P, Craig J. Consolidated criteria for reporting qualitative research

(COREQ): a 32-item checklist for interviews and focus groups. *International Journal for*

*Quality in Health Care*. 2007. Volume 19, Number 6: pp. 349–357

| **No. Item** | **Guide questions/description** | **Reported on Page #** |
| --- | --- | --- |
| **Domain 1: Research team and reﬂexivity** |  |  |
| *Personal Characteristics* |  |  |
| 1. Inter viewer/facilitator | BA and MK conducted the interviews | Page 6 |
| 2. Credentials | EC (MSc), BA (Bsc), MK (Bsc), ME (Diploma), PM (PhD), EN (Bsc), SN (Bsc), JM (MSc), AK (PhD), JN (PhD), AH (Prof), AA (Prof) | Author details page |
| 3. Occupation | EC (Doctoral fellow)  BA (RA)  MK (RA)  ME (RA)  PM (Post-doctoral fellow)  EN (Msc student)  SN (ECD technical assistant)  JM (Manager)  AK (Faculty)  JN (Lecturer)  AH (Prof)  AA (Prof) | Author details page |
| 4. Gender | Seven authors are female while 4 others are male | Author details page |
| 5. Experience and training | All the authors have experience in  qualitative research. EC, AK, JN and AA have vast experience supervising qualitative research projects | Author details page |
| *Relationship with participants* |  |  |
| 6. Relationship established | None |  |
| 7. Participant knowledge of the interviewer | None. The RAs introduced themselves to the participants during the interviews | Page 8 |
| 8. Interviewer characteristics | Yes. Table 1 | Participants description (table 1) Page 11 |
| **Domain 2: study design** |  |  |
| *Theoretical framework* |  |  |
| 9. Methodological orientation and Theory | Thematic approach  Bronfenbrenner’s ecological systems theory | Page 9  Page 6,7, and page 9, |
| *Participant selection* |  |  |
| 10. Sampling | Purposive and snowballing techniques | Page 7 |
| 11. Method of approach | The county facilitators shared the contacts of selected participants with the research assistants who then contacted them in advance to schedule the telephonic interviews at their convenient times | Page 7 |
| 12. Sample size | 103 interviews were conducted (68 key informants and 35 caregivers) | Page 7 |
| 13. Non-participation | None. | Page 7 |
| *Setting* |  |  |
| 14. Setting of data collection | The RAs conducted the interviews in private rooms at the workplace. The participants were encouraged to take the call in private and quiet places | Page 8 |
| 15. Presence of non-participants | None | Page 7 |
| 16. Description of sample | Participants’ socio-demographic data included age, gender, religion, residence, occupation, ethnicity, number of children, and the level of education. | Page 11 |
| *Data collection* |  |  |
| 17. Interview guide | The interview guide was pilot tested among 4 participants.  The interview guide is included in the Supplementary file 1 | Page 7, 8 and supplementary file 1 |
| 18. Repeat interviews | None | Page 8 |
| 19. Audio/visual recording | The interviews were audio-recorded | Page 8 |
| 20. Field notes | Interview summaries were done after the interviews | Page 8 |
| 21. Duration | 30-60 minutes | Page 8 |
| 22. Data saturation | During sampling, we strived to include a diverse sample of key stakeholders with varied levels of involvement with the early childhood development and ensured representativeness across the counties | Page 7 |
| 23. Transcripts returned | No. We anonymized all the transcripts | 8 |
| **Domain 3: analysis and ﬁndings** |  |  |
| *Data analysis* |  |  |
| 24. Number of data coders | 3 | Page 9 |
| 25. Description of the coding tree | Yes | Page 8-9 |
| 26. Derivation of themes | The themes were derived from the data | Page 9 |
| 27. Software | NVIVO QSR software Version 12 | Page 9 |
| 28. Participant checking | We did not conduct participant checking |  |
| *Reporting* |  |  |
| 29. Quotations presented | Yes | Page 12-26 and supplementary file 2 and 3 |
| 30. Data and ﬁndings consistent | Yes | Page 10-26 and supplementary file 2 and 3 |
| 31. Clarity of major themes | Yes | Page 10-26 and supplementary file 2 and 3 |
| 32. Clarity of minor themes | Yes | Page 10-26 and supplementary file 2 and 3 |
