# Supplementary material for: Intersection between individual, household, environmental and system level factors in defining risk and resilience for children in Kenya’s ASAL: A qualitative study
Source: PLoS One. 2025 Jan 17;20(1):e0316679. doi: 10.1371/journal.pone.0316679 (PMC11741590; doi:10.1371/journal.pone.0316679)
Supplement: S1 Table — (DOCX) [file pone.0316679.s003.docx]

**S1 Table: Supplementary table 1 on sources of risks**

| **Theme** | **KIIs (N)** | **Caregivers (N)** | **Total** | **Number of counties** | **Supporting quotes** |
| --- | --- | --- | --- | --- | --- |
| **Individual level factors (micro-level)** | | | | |  |
| Young mothers | 17 | 11 | 28 | 9 | ...Because if someone was married off at the age of 15, that person is a child. She doesn’t know anything about even the kids. She will just give birth probably for the sake, she does not know anything. So, age is a factor. *(Female, government official, 26 years)* |
| Older mothers | 1 | 3 | 4 | 4 | You know some of us give birth late, maybe like the age of thirty-seven, eight then forty. You know this child will grow up with a delay in mental development. *(Female caregiver, 33 years)*  Age of the mother if the mother was married if she was old, she may give birth to a disabled child. *(Female government official, 49 years)* |
| Illiteracy | 20 | 13 | 33 | 9 | Of course, the mother’s education level is low so she can’t even not even live when she goes to the hospital, they cannot read those instructions. She cannot read any other instruction in terms when they are given porridges you know manufacturers are providing how best you mix and provide this kind of food. So, if the mother cannot read obviously that child is affected in one way or the other. In terms of even food preparation, in terms of even understanding the child’s need. In terms of monitoring education at ECD level the mother has gap in all these areas. *(Male government official, 40 years)* |
| Mental health issues (stress) | 16 | 14 | 30 | 10 | Stress, if the mother is under stress, she cannot concentrate on what she is doing. Stress can make the mother neglect the children. She won’t care about them. *(Female caregiver, 40 years)* |
| Poor caregiver’s health | 7 | 8 | 15 | 7 | The issue of I think the health of the mother, when mother is sick, it's like the house stops functioning. So, and then most of the women are normally left alone, men go away with livestock. Everything looks at her. Actually, she is the husband and wife at the same time. And, when mother becomes sick or is poor, then things fall apart. *(Female caregiver, 33 years)* |
| **Family level factors (meso-level)** | | | | |  |
| Domestic violence | 13 | 17 | 30 | 9 | Domestic violence is one of the issues that complicates to divorces. It is one of the issues that complicates to child neglect, one of the issues that contribute to emotional abuse of the mother, and you know it’s very bad because domestic violence it can be at the expense of the child. They are cases where because of domestic violence the children are ignored so there so many cases at the children department because of the domestic violence. There are so many cases that are being handled by the elders of the village, religious leaders because of this domestic violence, these issues are coming because of polygamous marriages, its coming because of these issues of economic hardships and it is becoming because of this issue of drugs, domestic violence actually, largely contribute to neglect and abuse of the child. *(Female caregiver, 54 years)* |
| Drug abuse | 27 | 21 | 48 | 10 | Miraa chewers. Miraa is a very big problem here. So much in our community, some even end up preferring miraa more than alcoholism. Those parents that chew miraa they do not take care of their children. Yes, it’s a big issue. Sometimes you know mothers are being caned, they are beaten especially when these guys have chewed miraa and you know divorce is very high according to Islamic law the woman can ask for divorce and the man can also ask for divorce .so the divorce rate is high. Yes, which can lead to poverty. *(Male government official, 60 years)* |
| Poverty | 23 | 14 | 37 | 10 | Poverty. You sleep at night without food and when a child gets sick there is no money to take the child to the hospital, even to get fifty Kenyan shillings (<0.5 $). Children go to school without school uniform and bare feet. If there are no books, if there are no pens, or if there are no rubbers, if there are no shoes. Children form better off families use a raincoat when it rains but for the poor, they walk in the rain. So, poverty also contributes. *(Male government official, 62 years)* |
| Single parenting | 15 | 13 | 28 | 9 | Mother most of our parents are single mothers, they have children, but they are they don't have husband who are responsible, and they don’t have good jobs. So, finding something for the children to eat and to educate them is becoming a challenge. Because you have to feed them grow them (*(Male government official, 52 years)* |
| Lack of paternal involvement | 8 | 9 | 17 | 9 | When the child is around two year three years when they are playing is school, you can see the one with no father figure are mocked *(Male government official, 54 years)* |
| **Community and Socio-political factors (exosystem)** | | | | | |
| Insecurity | 13 | 5 | 18 | 8 | We also have a problem of insecurity that affects childhood outcome. Childhood outcome it’s all holistic about childhood education health and so on so you find this particular child in most cases where children are exposed to raids and banditry attacks and so on these particular children live in fear and worry for their life, and this in one way or another will affect even the way they learn at school, because at some point they have to move from one place to another with their parents *(Female Caregiver 29 Years)* |
| Political factors | 3 | 1 | 4 | 3 | County governments are corrupt and not so helpful *(Male caregiver, 38 years)* |
| **Socio-cultural factors (macro-level)** | | | | |  |
| FGM | 21 | 6 | 27 | 7 | Mother most of our parents are single mothers, they have children, but they don't have a husband who are responsible, and they don’t have good jobs. So, finding something for the children to eat and to educate them is becoming a challenge *(Male government official, 40 years)* |
| Early Marriages | 21 | 10 | 31 | 10 | Early marriages, you know the lady conceive and give birth at the age of sixteen and seventeen there is that low capacity in term of parental capacity. They are young and may not understand so any you know unexperienced. You know there is a lady who age birth at the age of twenty-five and the age of sixteen. These one at the twenty-five may understand a lot of things on her way, she has interacted with a parent or the community or neighbors and the children on how to take care of them. But a lady of as young as seventeen may not have found those experience. So, taking care of this child might to be outcome of a number of challenges with her. *(Male government official, 40 years)* |
| Nomadism | 18 | 6 | 24 | 9 | Parents do not pay much attention to education due to their nomadic way of life. Yes, many of them are nomads and the children to take care of the animals first until chiefs mobilize them to go back to school and are brought to school at 6 years or 7. So by this time this child has become over age and is not supposed to be in ECD classes. *(Female caregiver, 37 years)* |
| Polygamy | 16 | 7 | 23 | 9 | But all is not okay, there are some men who are fools. He will leave his first wife and marry another wife, then leave her and marry another one, then when the kids are many it’s obvious, he run away or decide to stay with one wife and leave the other three. But he cannot leave the marriage, he cannot divorce them, he will just leave it then the mother will struggle with the kids with hunger at home, generally the mother will be stressed together with her kids. *(Female caregiver, 42 years)* |
| Gender Bias and stereotypes | 17 | 5 | 22 | 8 | Now in this community the father is the decisions maker. Now, when the father is caring or has a knowhow on childcare on child devolvement it can affect the child either positively or negatively So majorly in this community the father is the decision making for example of a child is sick and the father decide that the child is to be taken the hospital is when the child is taken. Secondly, when the father, family planning is one of the factors that can contribute. And then there is good spacing of this child. The mother can have time to take care of the young ones and now we are doing here the benefit of the mother and children will involve the male. We do the male involvement which are father so that this it can affect the child indirectly. *(Male government official, 54 years)* |
| Food taboos | 8 | 2 | 10 | 6 | It is a practice, for example, there are other ideologies that children should not be given certain types of food like fish should not be eaten or certain vegetables, that is the ideology of the traditions of certain people. Now this is weakening the health of those children. *(Male government official, 70 years)* |
| Child labour | 7 | 4 | 11 | 8 | Let those old men take care of their goats and camels themselves but they are using a lot of child Labour. children who are supposed to be in school are looking after goats, they are looking after camels children who are 15 years have not gone to school So what we call perennial illiteracy is not going to leave us and this is the place that has the highest illiteracy rates and so something has to be done about that but this has not been done basically these are nomadic pastoralist keep shifting from place to place. *(Female government official, 64 years)* |
| Witchcraft | 2 | 0 | 2 | 2 | There are some diseases that people do not see the need to go to the hospital, for example epilepsy which is often related to evil powers. Now, when a child has such an illness, they are not treated, that is, they don’t take him to the hospital, they take him to traditional healers and witch doctors who do not help anything, and the situation is getting worse. *(Male government official, 70 years)* |
| Traditional ceremonies | 1 | 1 | 2 | 2 | The other challenge is social events. Whenever there are social events, most are attended by women. Women carry children with them and go with them. Absenteeism (in school) is the issue. A child can be absent for a week and come back after another week when parents are back. *(Male government official, 43 years)* |
| **Climatic and environmental factors (chronosystem)** | | | | |  |
| Drought | 25 | 14 | 39 | 10 | The pressure you have a family of six and you cannot feed them. The livestock have died. I will share with you what we experienced in the last 3 months. Devastating, people have lost almost everything especially livestock. Just coming the other day, it was Corona, after Corona it was drought, after drought came in the war, I think we do not know where and what we did wrong. (*(Male government official, 43 years)* |
| Heat stress | 22 | 12 | 34 | 10 | There are also environmental challenges whereby find sometimes it’s too hot so my child who is 8 months, so you find sometimes, they have a lot of sun burns, heat rash and so on but they kind get to adapt so those are some of the challenges that are there but being a resident of this particular environment, you get to cope with them and adapt. *(Female caregiver, 29 years)* |
| Wild animals attack | 11 | 1 | 12 | 6 | The only challenge maybe for taking to school, is when the schools are far. And then also sending a child to school which is about 2-3 km away, and the issue of wildlife insecurity. Because of this, they wait at home until they get at least past (the age) when they know how to go and come back on their own. So we realized that especially in rural areas, they delayed to go to school but these are isolated cases. *(Male government official 43 years)*  And along the lower region, we have a corridor, animal corridor, elephant corridor they normally move from Turkwel to Samburu along that corridor some are attacked by those animals, even schools along those area. *(Male government official ,40 years)* |
| Floods | 9 | 5 | 14 | 5 | You know sometimes there is small farming going on along the river and when the floods come all that is swept away and that’s when you get maybe communicable diseases like childhood diarrhoea, of course cholera, you know all those things. you know worms, that is when you get those things from floods. Displacement, no food, no settlement, you know you are exposed to diseases; malaria you know and many other diseases, yes. *(Male government official, 40 Years)*  Can you imagine a mother who is pregnant or lactating while displaced by floods? and IDP comes either in schools or IDP camp in a small shelter with five to six children. Mostly under ten and below. You can imagine that kind of scenario and the impacts they have on the mother. *(Male government official 40 years)* |
| Extreme cold | 6 | 5 | 11 | 5 | In fact, most of the time for now it’s almost four months’ due climate change has now shuffled a harsh common cold and fever and that now is going to cause climate changes and its affected mothers pregnant mothers of which now most them are sick because of this common cold they are feeling cold because now this cholera is there measles it depends on where you live by the moment and that is due to climatic change almost all manner of diseases. *(Male Caregiver, 41 years)*  Areas like these, the upper areas are very cold. And you find some families do not have enough shelter like blankets. Those areas are very chilly, children may be affected with problems of pneumonia, these respiratory infections. Like now in the highlands, the problem is that if you go to the hospital, many people have pneumonia, cough. Coughing so much. *(Male Caregiver ,70 years)* |
| Topography | 9 | 2 | 11 | 5 | Environmental degradation. The issue you know because of this climatic change and because of drought our land has now been degraded a lot. So, it not the even the grass and the crops are into germinating as it was before. And you know these animals only depends on grass and pasture apart from water. So, there are a lot of issue that are brought about by climate change in Garissa County. (M*ale government official ,40 Years*)  You see now maybe like for example road network is also another big issue. You find that people travel some distance without accessing any means of transport and I think there are also challenges in fact. *(Male government official, 62 years)*  We have incidences of rock falls in many of our parts since this place is rocky. Even it has affected almost 18 schools in the West Pokot that we have now reallocated our ECDE centers, around 18 of them*. (Male government official ,40 years)* |
| Wind and dust | 5 | 3 | 8 | 5 | You know our area is dusty and windy so most of them get some common cough and flu, you know maybe most children are not getting proper treatment, they also have some issues, is the challenge we live by. *(Male government official,43 years)*  Even some places in the rural setup, basically they are more desert. And now the wind is blowing like nobody else. We have s and even the children might not learn the whole day because of wind. *(Male government official 43 years)* |
| Lightening | 2 | 0 | 2 | 1 | Lightening, this one it’s also prone, 20% of West Pokot is prone to lightning strikes. *(Male government official ,40 years)* |
| Insect invasion | 1 | 0 | 1 | 1 | The official outbreak of locusts from Ethiopia they passed through Marsabit and to Wajir. Even the little that we had was wiped out. Green vegetation. And now the livestock were again exposed. (*Male government official, 43 years)* |
| **Biological factors** | | | | |  |
| Malnutrition | 23 | 13 | 36 | 10 | We have a lot of challenges when it comes to food, if you visit Marsabit today you will find a child who is 7 years old but resembles a 3-year-old child or a 4-year-old child because of food*. (Male caregiver ,46 Years)* |
