# Supplementary material for: Intersection between individual, household, environmental and system level factors in defining risk and resilience for children in Kenya’s ASAL: A qualitative study
Source: PLoS One. 2025 Jan 17;20(1):e0316679. doi: 10.1371/journal.pone.0316679 (PMC11741590; doi:10.1371/journal.pone.0316679)
Supplement: S2 Table — (DOCX) [file pone.0316679.s004.docx]

**S2 Table. Supplementary table 2 on sources of resilience**

| **Theme** | **KIIs (N)** | **Caregivers (N)** | **Total** | **Number of counties** | **Supporting quotes** |
| --- | --- | --- | --- | --- | --- |
| **Individual factors (micro-level)** | | | | |  |
| Hard work | 4 | 6 | 10 | 7 | Some children are resilient with the problems they have encountered. problems sometimes help them and with resilience a person sees that his parents are poor, they cannot help him, he can help him when he reaches a certain level, he understands the situation and tries to be firm so that he can change the situation so that it will be better. Perhaps he himself is trying to get a little money to fulfill his needs that way. Also, that makes them study hard, believing that if I study well, I can be lucky to change. *(Male government official ,70 years)*  Okay, if you see a child that raised in a difficult life, the child will understand that he needs a good life later, so a child that is raised in a dry place tends to concentrate a lot, wanting a good life ahead because he sees that the place he is in is not good. Yes, you often find that those children who have been sent to school are doing well because they remember what they go through at home. *(Female caregiver,38 years)* |
| Self-drive | 6 | 3 | 9 | 8 | You know a willing child will always manoeuvre his way and get out of the situation, so there are those children who have the will in them to achieve their dream, so when the children are encouraged, you find that they happen (to do well). *(Male government official, 52 years)*  That in fact is an individual factor we also have kids that are self-driven, you understand, like we have kids or that are self-driven and regardless of their family status. You know, like you've that inner drive. We've had kids in Marsabit succeeding because of that. *(Female government official ,38 years)* |
| Acceptance | 0 | 2 | 2 | 2 | Sometimes these children they accept. You know this issue, sometimes when you look at your family, you see that the economic situation is low, you see that there are many problems, you have to accept, you struggle and the little you have, you can achieve your target, that is, your future target. So there is to accept. *(Female caregiver ,37 year)* |
| **Family factors (meso-level)** | | | | | |
| Responsive caregiving | 7 | 9 | 16 | 7 | So, what about those with parental support? Maybe their parents try their best at home to give the child what he needs. Maybe the child has tuition at home, learning materials, books, internet may be, YouTube, Wi-Fi, he can be taught maybe, depending on how the parent is trying the best for his child. You will find that, maybe when a child leaves school he has another teacher at who coaches him at home, the parent is also supporting in terms of resources he needs. So, in that way you see the child is being provided with whatever he needs. *(Female caregiver, 29 years)* |
| Higher SES (socio-economic stability of the families) | 3 | 4 | 7 | 5 | Some parents have capacity to be able to care for their children. They have a capacity to take them to good school. They have the capacity to be take care of them nutritionally; they have capacity to be able to keep them in school. And of course the care, in terms of health and make sure that they pay school, they do not drop from school. It's about a family having a capacity to be able to care, care about their family and they have a responsibility of the both parents for their children, those are the children and they protect them from these other bad practices*. (Male government official ,40-50 years)* |
| Family discipline | 4 | 3 | 7 | 6 | When the parents are behaving well the children also get to copy their parents and they start imitating their parents and the whole community cares. *(Male government official, 45 years)* |
| Family advice | 4 | 4 | 8 | 5 | … having a mother who gives you advice. *(Female caregiver ,38 years)* |
| Caregivers hard work | 5 | 5 | 10 | 8 | …their efforts, parents are the ones who try hard. If there is a challenge, parents always try hard for their children to get it. *(Male caregiver, 38 years*)  ….. I know that if the child succeeds, it is the result of a parent trying hard to give support to his children, buy the necessary materials for school, feed his children, and that causes problems. A child close to his children. *(Male caregiver ,46 years)* |
| Education | 6 | 3 | 9 | 6 | The parents are well educated they know the importance of taking them to school. Talking about my children you know, those children apart from those below 3 years my children are graduates, education. You know telling these people to go to school. And anybody who went to school knows the value of school. *(Male government official 60 years)* |
| Mentorship | 2 | 0 | 2 | 2 | We have other teachers and parents who have been mentors. They encourage them and encourage the parents to encourage their children. I didn’t make it to this level but can at least try and manage. So, as much as they have the basic need, they have the food, they have the shelter, they have the clothing, they go to school and meet their teachers. And if the county has the qualified teachers they act as aspiration to them, so that they give them the desire to continue. So, I don’t think there are a lot of problems with children of you give them the necessary and the facility that are needed, we can nurture them you know a child is a blank book, you are the ones write on it, so it depends how you write on it. *(Male government official ,48 years)* |
| Coping to the environment | 11 | 5 | 16 | 8 | …you know despite challenges it’s like this our people have developed resilience. They have that resilience. They are used to this hardship. It’s like they are used to hardship you know everything from climate from the geographical set up including insecurity everything is hardship from early age these children are brought up in that way is like they have developed a kind of shock absorber. It’s like that resilience in a way. Then the major fact is that one of major factors that help some of the children now sustain and ignore all these challenges is resilience, cultural resilience, economic resilience. We are used to hardship so it’s like we have accepted to live with it so it has become part and parcel of us. So this child will grow they will be part of school especially local schools where there is no teachers where there is no resources but still you will find child getting up getting 400 points on to find a child getting A- so its resilience and accepting so we have accepted our situation. We have accepted that this is our life and life is a factor which actually gives us a boost. *(Female caregiver, 54 years)* |
| **Community and Socio-political factors (exosystem)** | | | | | |
| Financial support (bursaries) | 3 | 3 | 6 | 5 | There are projects that provide us with cash transfers. You will probably get Ksh.5000, after every two months. They use that money to exchange money for their children and I have seen many families who at least use that money to buy some food for their children that they usually don't buy. They even eat during the day and also eat in the evening, so they contribute a lot. The mothers try very hard to contribute at least to change the past life which is challenging until normal now. (*Male caregiver, 46 years)* |
| Provision of health services | 3 | 2 | 5 | 3 | So, the parents have some local skills they get from local nurses, at home level each mother and each parent will have to try and look at the health of the child. But at the community level we have community centres, health centres which play a large part in the health of a child. *(Male caregiver, 54 years)*  And regular immunization as the government schedule. Another one is also periodic immunization where if there is an outbreak there is immunization. All those are some of the things that the government does that support maternal and young child and newborn programs. *(Male government official ,43 years)* |
| CSO support (nutrition, education, and community empowerment) | 5 | 1 | 6 | 3 | And civil Society activities that have helped them to be resilient. The information we provide empowers people to make better decisions and then support from the government, really, we do not have overlook it. It's fantastic because the government is the key stakeholder and duty bearer. Though they have not done their part so well but they have managed much better than the way it used to be previous years*. (Male government official ,43 years)*  There is one of our stakeholders called MWADO. They work for women rights organization. They have taken so many people to departments of children, courts, assisting them. Somehow men are being compelled to take up their man roles by force, to decide their responsibility. *(Male government official, 43 years)* |
| **Socio-cultural factors (Macro-level)** | | | | |  |
| Community support and cohesion | 11 | 6 | 17 | 9 | Community members who mobilize themselves to see they address the issue of education for the children. There is some case where the parents are very much concerned so they come together and look at how they can improve there, the education situations. They even pay the teachers sometimes something like that. *(Male government official ,43 years)* |
| Community role models | 5 | 5 | 10 | 6 | I grew up talking to other people, who have done well in the community who have been better people in the community and there are those inspirations that people have come to see. You know when you win and achieve your goals and a come back to the society to give back. So, they have done those things and these children they are willing and they aspire to be more than what their parents were*. (Male government official ,48years)* |
| Community discipline | 5 | 1 | 6 | 5 | …We have some community structure where we have the leadership in the community, they come together they form a structure here we have the elderly in place. Then now the elderly look at issue that are brought to them maybe there is decision making as open of the parent is involved. Most cases you find that they discuss issues on leadership they discuss issues on child offences. Like somebody if you have done any offence you have done through theft, you have stealing you are taken there and they make a decision. So, if a parent is involved there, the parents will teach the child that it is wrong to do this if you are taken to that structure those people you will punishment and maybe you will be an outcast and a decision will be taken upon you. So that one will make children to grow properly though they will be looking forward if the is a structure in place that give leadership on matters that happen in the community. Before anything is done is that committee that will approve whatever to happen and it will. If it disapproves it will not happen. *(Female government official ,25 years)* |
| Deviation from harmful cultural practices | 3 | 0 | 3 | 2 | We are also slowly moving out of dangerous and retrogressive cultures. Previously, when a child for example gets pregnant when in school, she drops out of school all together. But nowadays they go back to school and finish their education. So it is another opportunity. So you can see girls they have babies, they leave babies back at home and they return to school and fight them to the degree level. All these you know; all these are giving people like hope. To succeed, where there is adversity*. (Female government official ,38 years)* |
| Religion | 15 | 11 | 26 | 9 | As I have said earlier you see some of these social factors, like Islamic festivals and other festivals that are there some they build. You know when you talk of (Islamic term), people are insisted in learning behavior of the prophet, people are insisted in following the manners of prophet you see the life and the history of the prophet so it gives them a good motivation and a good inspiration indeed. *(Male caregiver, 30 years)* |
| **Biological factors** | | | | |  |
| Breastfeeding | 4 | 1 | 5 | 4 | …the religion encourages the children to be breast feed up to 2 years or more*. (Female government official ,49 years)* |
| Immunization | 4 | 0 | 4 | 4 | The public health teams go around vaccinating children below five years, this one is also boosting their immune, others are also providing food supplements because their mothers are not producing enough milk because they are not eating well, so they are provided with supplements that has stronger vitamins components and other protective minerals. These ones are also coming in. Then other than that, the issue of maybe maternity leaves, people staying with their families for the first three months, is also boosting, also the mother just be with their little one and breastfeeding. *(Male government official ,43 years)*  You know, in the last 3 years, this deworming, as I tell you the uptake of immunization has increased 28% pre-devolution to 55%. And there is also continuous growth monitoring at the health facility. There is also that, they call it, infant, newborn and mother nutrition approaches. There is another protocol that you integrate maternal, young child and newborn. And there is this program, call it “malezi bora” also. That the community health workers normally the households to teach them on good feeding practices. To ensure there is exclusive breastfeeding for the first 6 months. And when weaning comes, they wean appropriately. *(Male government official, 43 years)* |
| Good nutrition | 4 | 3 | 7 | 5 | In terms of capacity building, teachers convincing parents, community members and sometimes providing supporting feeding programs like the world food program. And even the relief food helps the parents at the rural areas. *(Male government official ,43 Years)* |
